# Simultaneous Recovery of Magnesium and Lithium from Salt Lake Brine by Membrane Electrolysis for Resource Utilization

**DOI:** 10.3390/ma18225077

**Published:** 2025-11-07

**Authors:** Xijuan Pan, Jingyu Jia, Yu Han, Wencheng Li, Xiang Li

**Affiliations:** 1 Beijing Key Laboratory of Theory and Technology for Advanced Batteries Materials, School of Energy Storage Science and Engineering, North China University of Technology, Beijing 100144, China; 2 Beijing Laboratory of New Energy Storage Technology, North China University of Technology, Beijing 100144, China

**Keywords:** high Mg/Li ratio, electrolysis temperature, separation and extraction, cationic membrane electrolysis

## Abstract

The extraction of lithium and potassium from salt lakes has led to the generation of substantial amounts of magnesium-rich waste streams. These by-products, with their high magnesium content, have contributed to severe environmental degradation in salt lake regions. Therefore, recovering and utilizing magnesium from salt lake resources is a crucial challenge for achieving sustainable development. In this study, magnesium and lithium were separated from evaporated brine—obtained via solar pond technology—using membrane electrolysis. Magnesium was converted into Mg(OH)_2_ as a flame retardant, while lithium was refined into battery-grade Li_2_CO_3_. The final products exhibited high purity, exceeding 99.5% for Mg(OH)_2_ and 99.99% for Li_2_CO_3_. This work systematically investigated the influence of electrolysis temperature on the physicochemical properties of Mg(OH)_2_ extracted via membrane electrolysis. The variation in electrolyte temperature was also analyzed in relation to other process parameters, such as electrolyte concentration, current density, and processing time. Results demonstrated that the electrolysis process could maintain a favorable operating temperature through self-heating, even under ambient conditions. Using this electrolysis approach for magnesium–lithium separation from brine, extraction rates of 95.86% for magnesium and 67.46% for lithium were achieved.

## 1. Introduction

Salt lakes represent significant liquid reservoirs rich in various metallic resources. After decades of development, they have become a primary source for elements such as lithium, magnesium, potassium, and boron [1,2,3,4]. Therefore, the rational and eco-friendly exploitation of these resources is critically important.

Being a major agricultural country yet facing a shortage of potash resources, China initiated its salt lake development efforts with potash production [5]. Historically, nearly half a century of potassium extraction from salt lakes led to the discharge of large amounts of magnesium-rich waste liquids and the accumulation of low-grade magnesium salt products, due to limited economic development and insufficient environmental awareness at the time [6,7]. In contrast, the advancement of lithium battery technology has generated substantial market demand for lithium, significantly accelerating the development of lithium extraction processes from salt lakes [1,8]. The extraction of lithium alone has resulted in the same issue previously seen with potassium production: the accumulation of vast amounts of magnesium-rich waste. This substantial waste of magnesium resources is largely due to China’s abundant magnesium ore reserves and mature smelting technology, which diminish the economic incentive for magnesium recovery from salt lakes. Consequently, the stockpiling of large volumes of magnesium-rich solid and liquid waste not only occupies significant land—often encroaching on mineral deposits—but also causes severe environmental contamination. For instance, current potash production in Qinghai generates approximately 120 million cubic meters of magnesium-containing waste annually, which consumes considerable storage capacity and contaminates nearby potassium chloride deposits [9]. In light of increasing environmental awareness and stringent policy requirements, the sustainable management of magnesium-containing waste streams from potash and lithium production has become an urgent challenge that must be addressed.

Most reported methods for treating salt lake brine focus on the separation and recovery of a single element, such as lithium, potassium, or boron. A common approach involves solar pond evaporation to concentrate the brine, remove sodium chloride and partial magnesium sulfate, and then introduce lime for calcium and magnesium removal. However, as most salt lakes in China feature high Mg/Li ratios, this magnesium removal process generates significant magnesium-rich waste. In recent years, various new separation and extraction technologies have been progressively refined and put into practical application, such as adsorption methods, extraction methods, and electrochemical methods. Adsorption technology can be further divided into ion sieves, adsorption membranes, electrochemical adsorption, etc. Metal-based adsorbents exhibit a high degree of technological maturity, though their adsorption capacity still has room for improvement. Adsorption membranes offer high selectivity but are limited in their number of reuse cycles. Among them, electrospun nanofibrous membranes possess a higher specific surface area and porosity, but their mechanical strength is relatively poor. All the aforementioned methods require the use of acid to desorb lithium. In contrast, electrochemical adsorption utilizes electricity instead of acid, thereby significantly reducing chemical reagent consumption [10]. Heidari et al. [11] achieved a lithium ion (Li^+^) adsorption efficiency (percentage of lithium removed from the solution by the solid adsorbent) of 76.4% using aluminum chloride (AlCl_3_∙6H_2_O) as an adsorbent. Similarly, Jia-Li Xiao et al. [12] reported that a polyacrylamide–MnO_2_ ion sieve exhibited high selectivity for Li^+^ recovery from brine. Using TBP as the extractant, Dong Shi et al. [13] achieved a lithium extraction efficiency (percentage of lithium transferred from the initial brine phase to the organic phase) of 87.9% with a corresponding decrease in the Mg/Li mass ratio from 370 to 0.02. N. Beyond adsorption, electrochemically driven membrane technologies have also garnered increasing attention for the separation and extraction of valuable elements from salt lake resources [14,15,16,17,18]. It was demonstrated by Xiao-Yao Nie et al. [19] that monovalent selective ion-exchange membranes could achieve a 95.3% recovery of Li^+^ while lowering the Mg/Li mass ratio from 150 to 8.0. A key limitation of these unit-process methods, however, is the lack of holistic brine treatment and the potential secondary pollution from organic reagents.

Salt lakes are classified into various types based on their distinct composition and concentration, such as magnesium sulfate type, sodium sulfate type, high-carbonate type, chloride type, and moderate-carbonate type. This paper primarily investigates the magnesium sulfate type. The ionic composition and concentration ranges of the raw salt lake brine and the concentrated solution obtained via solar pond evaporation are presented in Table 1 [3,20,21,22,23]. Magnesium and chloride were the predominant cation and anion, respectively, while lithium and boron were also significantly enriched.

Electrochemical membrane technology, which has been rapidly advancing, offers an effective approach for separating magnesium, lithium, and boron from salt lake brine. Building on previous studies [3,20,21], this work further investigates the extraction and separation of magnesium and lithium via membrane electrolysis, aiming to produce high-purity magnesium hydroxide for use as a flame retardant and battery-grade lithium carbonate. The influence of electrolysis temperature on the physicochemical properties of the magnesium hydroxide product and on the electrolytic process was systematically examined. In addition, the effects of other key electrolysis parameters—including anolyte and catholyte concentrations, current density, and electrolysis time—on the system temperature during operation were also analyzed.

## 2. Materials and Methods

### 2.1. Experimental Materials

The magnesium chloride solution, the lithium chloride solution, and the simulated mixed brines were all prepared in-house. The analytically pure reagents (MgCl_2_·6H_2_O, LiCl, and MgSO_4_·7H_2_O) used in this experiment were supplied by Tianjin Kermel Chemical Reagent Co., Ltd. (Tianjin, China). The analytically pure KCl and Na_2_B_2_O_7_·10H_2_O were bought from Sinopharm Chemical Reagent Co., Ltd. (Shanghai, China). The cathode employed in the electrolysis experiments consists of a pure titanium plate, whereas the anode comprises a titanium plate coated with noble metal oxides (Ir_2_O_3_ and Ru_2_O_3_). The effective area of each electrode plate is 7.5 cm^2^, and the volume of electrolyte in each chamber is 300 mL. The cationic membrane was bought from Hefei ChemJoy Polymer Materials Co., Ltd. (Hefei, China), and its performance parameters are presented in Reference [3].

### 2.2. Preparation of Mg(OH)_2_ as a Flame Retardant

**Figure 1:**
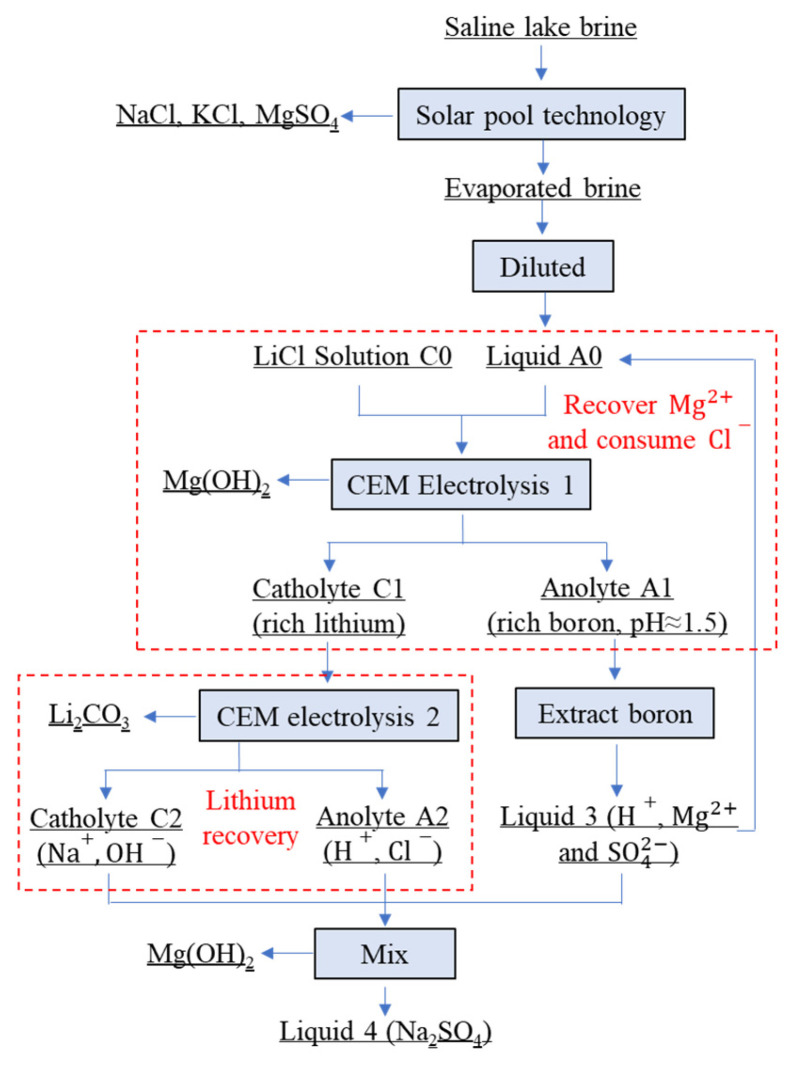
Flowchart for the separation and extraction of magnesium and lithium from brine [3].

This study initially conducted fundamental research on membrane electrolysis experiments using magnesium chloride solutions, investigating the impact of electrolyte temperature on the electrolysis process and examining the influence of other electrolysis parameters on the system temperature. Subsequently, membrane electrolysis was employed to separate magnesium from brine, utilizing diluted simulated salt lake brine (evaporated via solar pond technology) as the anolyte and lithium chloride solution as the catholyte. Battery-grade lithium carbonate was obtained by employing the post-magnesium-separation electrolytic catholyte as the electrolyte for lithium extraction. The detailed process flow is illustrated in Figure 1. For the magnesium and lithium recovery cycles shown in Figure 1, we define the extraction rate as the percentage mass reduction in ions in the electrolyte, calculated by comparing concentrations before and after the process.

The electrochemical reactions involved in the cationic membrane electrolysis process for magnesium–lithium separation are primarily chlorine evolution at the anode and hydrogen evolution at the cathode, as represented by Equations (1) and (2), respectively. The hydrogen gas generated at the cathode serves as a valuable by-product with extensive applications in clean energy fuels, industrial chemical synthesis, medical applications, and metallurgical processes. Similarly, the chlorine gas produced at the anode constitutes an important by-product widely utilized in water disinfection, organic compound synthesis, chlorometallurgy, and various other industrial applications. The comprehensive electrochemical reactions governing magnesium separation are depicted in Equations (3) and (4), while the reactions associated with lithium carbonate purification are illustrated in Equations (5) and (6). Following filtration and phase separation, the precipitate products obtained from the catholyte solution were subjected to air-drying at 70 °C for a period of 8 h.

(1) $$2{Cl}^{-}-2e\to {Cl}_{2}↑$$

(2) $$2H_{2}O+2e\to {2OH}^{-}+H_{2}↑$$

(3) $${Mg}^{2+}+{2OH}^{-}\to {Mg(OH)}_{2}↓$$

(4) $${MgCl}_{2}+H_{2}O\overset{electrify}{\to }{Mg(OH)}_{2}↓+H_{2}↑+{Cl}_{2}↑$$

(5) $${{2Li}^{+}+2OH}^{-}+{CO}_{2}={Li}_{2}{CO}_{3}↓+H_{2}O$$

(6) $$2LiCl+H_{2}O+{CO}_{2}\overset{electrify}{\to }{Li}_{2}{CO}_{3}↓+H_{2}↑+{Cl}_{2}↑$$

### 2.3. Analysis Methods

#### 2.3.1. Physicochemical Properties and Morphology Analysis

The phase composition of the electrolysis products was analyzed using X-ray diffraction (XRD, D8 Advance, Bruker, Berlin, Germany). The microstructure morphology and particle size distribution were characterized by scanning electron microscopy (SEM, SU8010, Hitachi, Tokyo, Japan) and laser particle size analysis (Mastersizer 3000, Malvern, Worcestershire, UK), respectively. The concentrations of metal ions (Mg^2+^, Li^+^, Na^+^, and K^+^) in the solution were quantified via inductively coupled plasma optical emission spectrometry (ICP-OES, Optima 8300 DV, PerkinElmer, Waltham, MA, USA), while the anionic species were determined by ion chromatography (IC, 930 Compact IC Flex, Metrohm, Herisau, Switzerland). Due to its relatively high concentration, the sulfate ion (SO_4_^2−^) content in the anolyte was measured using a chemical precipitation method.

The XRD (D8 Advance X, Bruker, Germany) was used to analyze the phase of electrolysis products. The micro morphology and particle size distribution were detected by SEM (SU8010, Hitachi, Japan) and Laser Particle Sizer (Mastersizer3000, Malvern, UK). The ICP–OES (Optima 8300DV, Perkin–Elmer, Waltham, MA, USA) was used to determine the contents of metal ions (Mg^2+^, Li^+^, Na^+^, and K^+^) in solutions. And the anions were measured by IC (930 Compact IC Flex, Metrohm, Switzerland). The SO_4_^2−^ concentration in anolyte was detected by the chemical precipitate method because of its higher concentration.

#### 2.3.2. Data Analysis

The detailed methods for calculating current efficiency and unit energy consumption can be found in previous works [16,20,21], with the corresponding formulas given in Equations (7) and (8).

(7) $$\eta =\frac{NF\cdot n_{{Mg(OH)}_{2}}}{I\cdot t}\times 100\%$$

(8) $$W=\frac{\int_{0}^{t}UIdt}{m_{{Mg(OH)}_{2}}}$$

where η is the current efficiency (%), N is the number of electrons transferred per mole of products (2), F is the Faraday constant (96,485 C/mol), $n_{{Mg(OH)}_{2}}$ is the mole of magnesium hydroxide products (mol), *I* is the applied current intensity (A), *t* is the time of electrolysis (s), *U* is the cell voltage (V), and $m_{{Mg(OH)}_{2}}$ is the mass of the electrolysis product magnesium hydroxide (g).

## 3. Results and Discussion

### 3.1. Effect of Electrolyte Temperature on Physicochemical Properties of Products and the Electrolysis Process

The cathode chamber and anode chamber on both sides of the cationic exchange membrane were, respectively, 70 g/L and 150 g/L magnesium chloride solutions, with a volume of 300 mL, a current density of 335 mA/cm^2^ (current of 2.5 A), and an electrolysis time of 2 h. When the electrolyte temperature is 20, 35, 50, 65, and 80 °C, the physical and chemical properties, like phase, morphology, and particle size of the precipitated products by membrane electrolysis, are analyzed. The influences of different electrolyte temperatures on cell voltage, pH value of cathode solution, current efficiency, and energy consumption per unit mass of magnesium hydroxide were also investigated. The electrolytic experiments were carried out in a constant temperature water bath. The experiment at 20 °C was cooled by ice water to ensure a stable temperature in the electrolyte.

#### 3.1.1. Effect of Electrolyte Temperature on Product Physicochemical Properties

**Figure 2:**
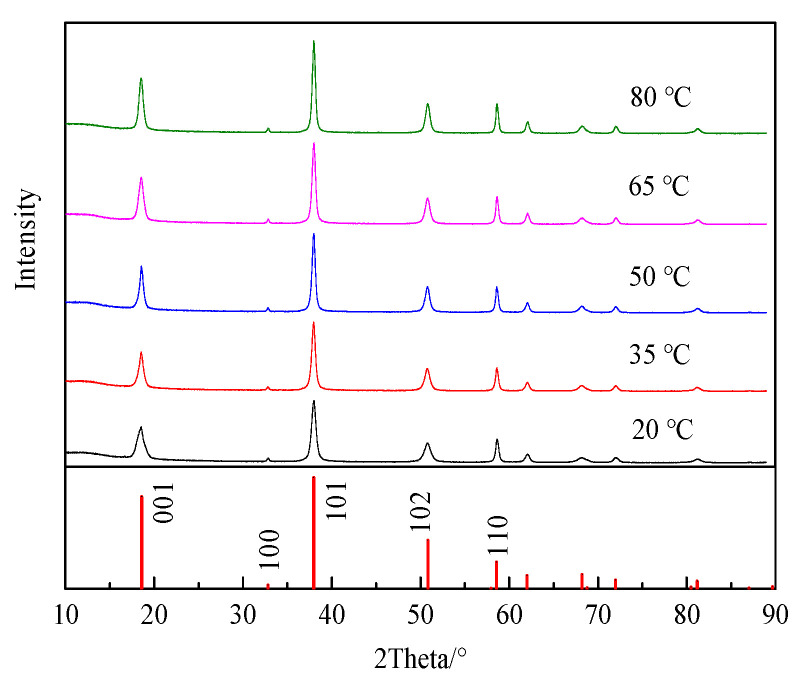
XRD patterns of the electrolysis products obtained at different electrolyte temperatures.

The electrolytic products prepared at different temperatures were tested on a powder X-ray diffraction analyzer, and the results are shown in Figure 2; XRD patterns show that the electrolytic product is magnesium hydroxide without an obvious impurity diffraction peak. The intensity of the diffraction peak increases with the temperature of the electrolyte. Magnesium hydroxide has five major diffraction peaks, with (001) representing the nonpolar faces of the crystal and (101) and (110) representing the polar faces of the crystal.

**Figure 3:**
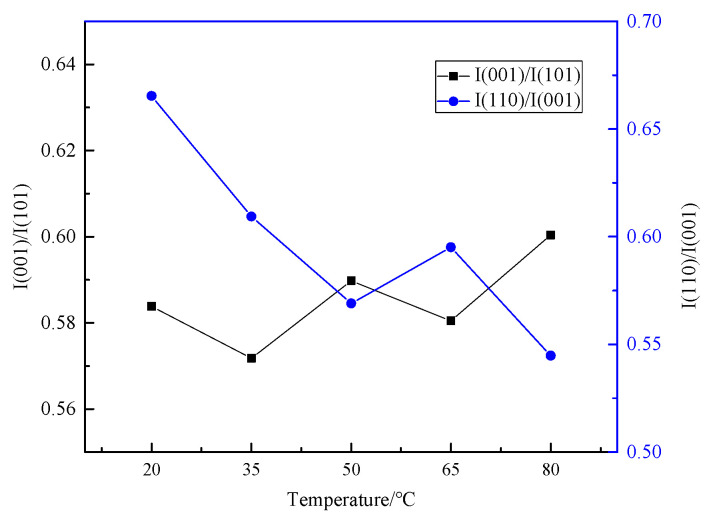
Effect of electrolyte temperature on the intensity ratio of the Mg(OH)_2_ X-ray diffraction peak.

Figure 3 shows the X-ray diffraction peak intensity ratio (I001/I101) of non-polar face (001) to polar face (101) and the ratio (I110/I001) of the intensity of polar face (110) to non-polar face (001). As the temperature increases, the intensity ratio (I001/I101) increases on the whole, while the ratio (I110/I001) decreases. This indicates that a higher electrolyte temperature is beneficial to the growth of the magnesium hydroxide crystal non-polar face (001) and the production of magnesium hydroxide with higher dispersion. The lower electrolyte temperature is more conducive to the growth of crystal polar faces (101) and (110). The dispersion of the obtained products is poor, the polarity of the products is enhanced, and the agglomeration phenomenon is easy to occur.

**Figure 4:**
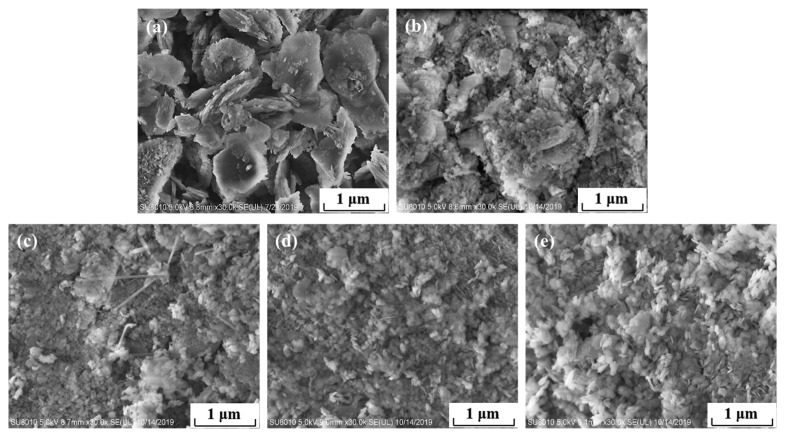
SEM images of the products synthesized at different electrolyte temperatures. (**a**) 20 °C; (**b**) 35 °C; (**c**) 50 °C; (**d**) 65 °C; and (**e**) 80 °C.

Figure 4 shows the micromorphology of magnesium hydroxide prepared at different electrolyte temperatures. As can be seen, there are three main types of magnesium hydroxide crystal monomers: needle-like, large-size round sheets, and nanoscale, small round sheets. The monomers of magnesium hydroxide prepared at 20 °C are mostly micron-sized round sheets. At 35 °C, it is mainly composed of nanoscale small wafers, including a small number of large laminar particles. The micromorphology of magnesium hydroxide prepared at 50 °C showed a nanoscale wafer and a small number of long needle-like particles. The electrolytic products at 65 °C and 80 °C are mainly nanometer-sized round flake particles.

**Figure 5:**
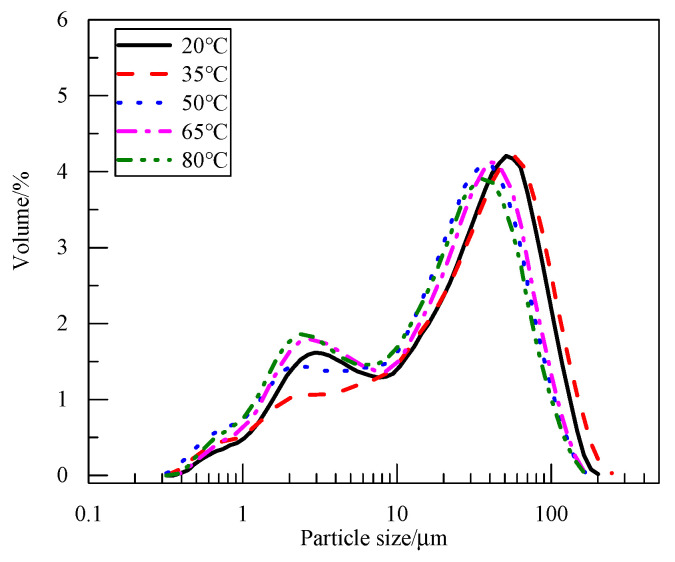
Particle size of products synthesized under different electrolyte temperatures.

Figure 5 shows the particle size distribution curve of the electrolytic product magnesium hydroxide at different temperatures. Electrolytic products have two main particle size ranges, 2~3 μm and 40~60 μm. As can be seen from the SEM micrograph, the primary particle size of magnesium hydroxide is very small, at <3 μm. However, during the process of cathode liquid filtration and cake drying, the particles of magnesium hydroxide were agglomerated. The particle size measured by the laser particle size meter was the secondary particle size after the breakdown of electrolytic products, and the particle size increased significantly. Because the polarity of electrolytic products is different, the degree of agglomeration is also different. With the increase in electrolyte temperature, the non-polarity of electrolytic products increases, the agglomeration degree decreases, and the particle size decreases.

#### 3.1.2. Effect of Electrolyte Temperature on Parameters

The temperature of the electrolyte has a great influence on the properties of the electrolyte. The ionization degree, hydrolysis degree, ion migration rate, reaction rate, and resistance of solute in electrolyte are all affected by the temperature. Therefore, it is of great significance to study the influence of electrolyte temperature on cell voltage and pH of the cathode solution. In addition, the current efficiency and unit energy consumption of electrolysis products can also play a guiding role in the study of the membrane electrolysis process.

**Figure 6:**
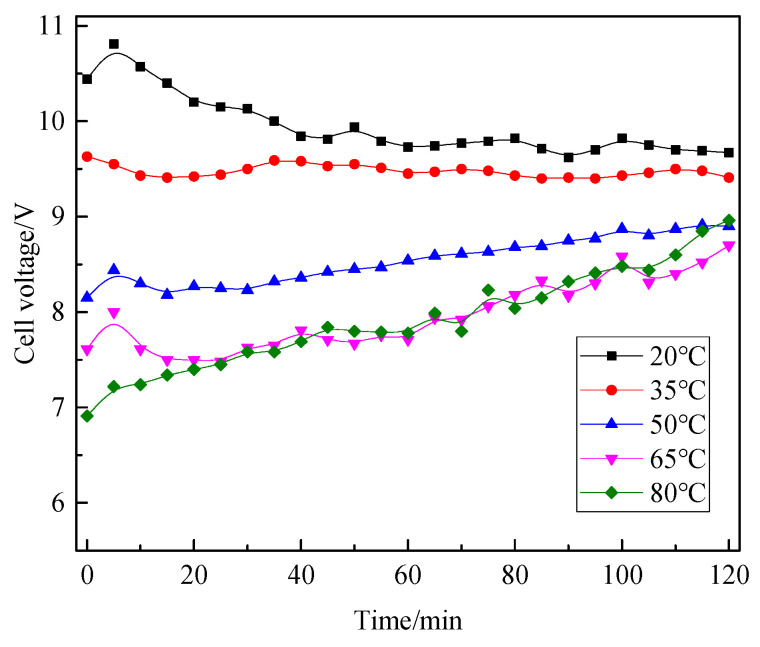
Variation in cell voltage under different electrolyte temperatures during electrolysis.

The initial (time of 0) cell voltages of electrolysis were indicated in Figure 6. The higher the electrolyte temperature, the lower the initial cell voltage. The increase in temperature promotes the ionization of magnesium chloride in the electrode liquid, improves the movement rate of ions in the solution, thus enhancing the conductivity of the liquid, reducing the resistance of the solution, and thereby reducing the cell voltage. The change in electrolyte temperature will also affect the membrane resistance of the cationic exchange membrane between the anode and cathode. The increase in temperature will reduce the membrane resistance and promote a decrease in the cell voltage.

When the electrolysis time was <5 min, there was a significant increase in the cell voltage, which soon recovered to the corresponding voltage value of the overall change trend. This is because at the beginning of electrolysis, the electrode plates were just immersed in the electrode liquid, and the contact between the solid and liquid phases was not very good. There may be tiny bubbles on the electrode plate surface, and the contact resistance between the two phases is relatively large. With the extension of time, the electrode surface is completely wetted, and the contact resistance gradually decreases to a stable value, which no longer affects the voltage in the electrolysis process, and the value of the cell voltage gradually returns to the overall change trend.

When the electrolyte temperature is lower than 35 °C, the cell voltage gradually decreases with time and remains stable until the end of electrolysis. In the process of electrolysis, the voltage of the electrolyte at low temperature is always higher than that of the electrolyte at high temperature. When the electrolyte temperature is higher than 50 °C, the cell voltage increases with time until the end of the electrolytic experiment. When the electrolyte temperature was higher than 65 °C, the cell voltage in the electrolytic process generally increased, but the cell voltage in the two groups of electrolytic experiments was not stable. The small change in voltage may be due to the local concentration polarization of electrolyte between the two electrode plates, which leads to the fluctuation of solution resistance and the fluctuation of cell voltage.

**Figure 7:**
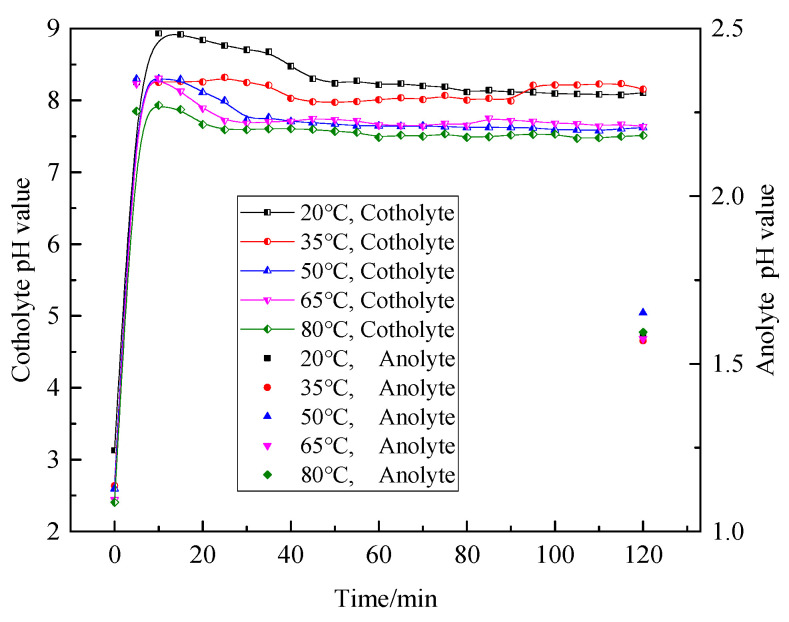
Variation in pH under different electrolyte temperatures during electrolysis.

Figure 7 shows the variation in catholyte pH value and anolyte pH value at the end of electrolysis at different electrolyte temperatures. The increase in cathode liquid temperature promotes the ionization of magnesium chloride and the hydrolysis of Mg^2+^; thus, the pH value of cathode liquid gradually decreases, while the increase in acidity promotes the increase in conductivity. Therefore, the higher the temperature of the electrolyte, the lower the pH value in the electrolytic process. As the concentration of magnesium chloride gradually decreased, the influence of temperature on ionization gradually decreased, and the difference in pH values of different temperatures also gradually narrowed. The range of pH value of the cathode solution with different electrolyte temperatures was narrowed from 7.5~8.7 at 20 min of electrolysis to 7.5~8.2 at the end of electrolysis. The pH value of the cathode solution was rapidly increased to 7.8~8.8 owing to the hydroxide ion generation. With the migration of Mg^2+^ from the anode solution to the cathode solution and the precipitation with hydroxide ions, the pH value of the cathode solution decreased slightly and finally stabilized in the range of 7.5~8.5. The terminal pH value of the anode liquid was not affected by the temperature, which was about 1.6, but only slightly lower than 50 °C, which was about 1.7.

**Figure 8:**
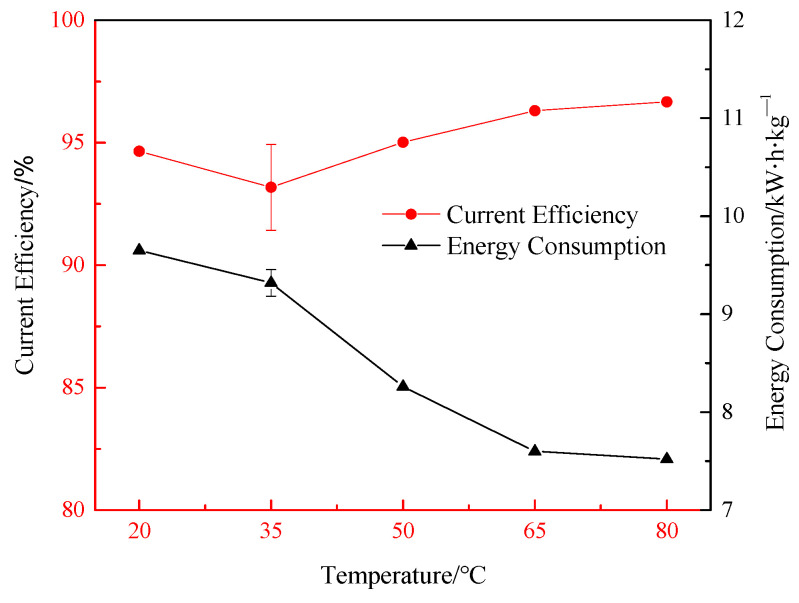
Effects of electrolyte temperature on current efficiency and energy consumption.

Figure 8 indicates the influence of electrolyte temperature on current efficiency in the electrolysis process and energy consumption of magnesium hydroxide product per unit mass. The increase in electrolyte temperature can reduce the cell voltage, improve the current efficiency, and reduce the energy consumption. As the electrolyte temperature increased from 20 °C to 80 °C, the current efficiency increased from 94.65% to 96.67%, and the energy consumption per unit mass of magnesium hydroxide decreased from 9.65 kw∙h∙kg^−1^ to 7.52 kw∙h∙kg^−1^, resulting in a 22.02% decrease in energy consumption.

Although it takes extra energy to keep the electrolyte at a high temperature, the process itself generates heat to help maintain the temperature. In addition, the experimental equipment of the laboratory scale is small, and the heat loss is high. The large electrolytic equipment in industrial production loses heat much more slowly, and the temperature of the electrolytic system, which can be maintained by electrolysis itself, is higher than that measured in the experiment in this paper. Therefore, considering the influence of current density on operating parameters, energy consumption parameters, and physico-chemical parameters of electrolytic products in the electrolytic process, 50 °C was selected as the optimal electrolyte temperature in the laboratory electrolytic experiment, while the electrolytic temperature in the industrial experiment could be considered as 80 °C.

### 3.2. Effect of Different Experimental Conditions on the Temperature Change During Electrolysis

**Figure 9:**
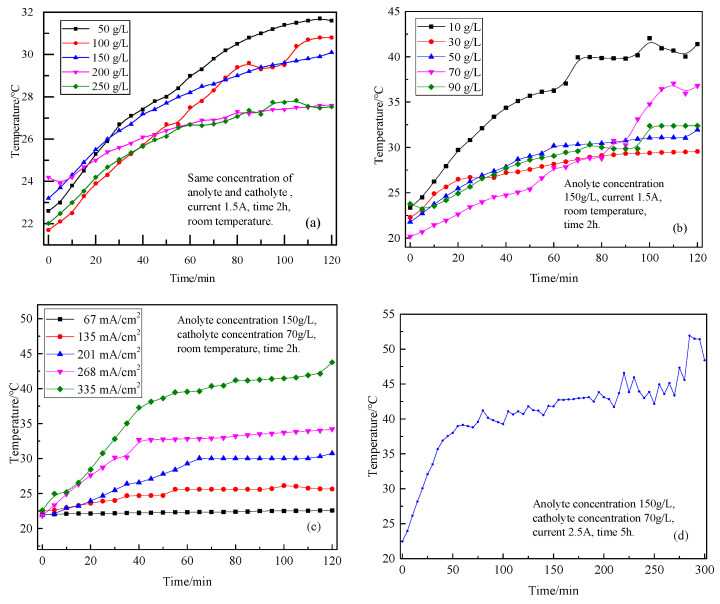
Effects of different conditions on electrolyte temperature during electrolysis process. (**a**) Electrolyte concentration, (**b**) catholyte concentration, (**c**) current density, and (**d**) electrolysis time.

Because the resistance of the electrolytic system cannot be eliminated, the electrolytic process will always be accompanied by the generation of heat, so the electrolyte temperature will be affected by other electrolytic parameters. In this paper, the influence of the concentration of two electrolytes, current density, and electrolysis time on the temperature of the electrode liquid in the electrolysis process was investigated. The various curves of electrolyte temperature with electrolysis time are shown in Figure 9.

Figure 9a shows the influence of different electrolyte concentrations on the temperature of the electrode liquid. Due to the resistance in the electrolytic system (solution resistance, electrode resistance, contact resistance, membrane resistance, etc.), some electric energy is always converted into heat energy in the electrolytic process, which makes the electrolyte temperature rise. When the electrolysis time reaches 2 h, the cathode liquid temperature rises to about 30 °C. When the concentration of MgCl_2_ in the electrolyte was below 200 g/L, a higher concentration led to increased ion availability, thereby enhancing the solution’s conductivity. This resulted in lower electrical resistance, reduced Joule heating during electrolysis, and consequently, a lower electrolyte temperature. When the electrolyte concentration is 250 g/L, the temperature curve of the cathode liquid after 1 h of electrolysis basically coincides with that of electrolyte concentration of 200 g/L. This indicates that when the concentration of electrolyte is higher than 200 g/L, the conductivity of electrolyte no longer increases with the concentration of electrolyte. Interionic interaction and solution viscosity increase the movement resistance of ions and affect the conductivity of the solution.

The temperature changes during electrolysis of the cathode solution with different concentrations of magnesium chloride are shown in Figure 9b. Since heat is always generated during electrolysis, the temperature of the electrolyte increases. When the temperature rises to a certain value, the heat generated by electrolysis and the heat lost by the system reach a balance, and the electrolyte temperature will no longer rise, maintaining a small range of fluctuations within a temperature range. The lower the concentration of the cathode solution, the lower the concentration of ions in the solution, the greater the resistance of the solution, and the more heat generated in the electrolysis process. Therefore, the lower the concentration of the cathode liquid, the faster the temperature increase of the cathode liquid in the electrolysis process, and the higher the final equilibrium temperature.

The temperature change in the cathode solution during electrolysis with different current densities is shown in Figure 9c. The current density directly determines the current and, therefore, the amount of heat produced. When the current density is 335 mA/cm^2^, the heat generated by the system is always more than the heat lost, so the cathode liquid temperature continues to rise. However, with the increase in the temperature of the system, the temperature difference between the system and the surrounding environment becomes larger and larger, the heat is lost faster, and the temperature of the electrode liquid increases slowly, so the slope of the temperature and time curve becomes smaller and smaller. When the current density is 135~268 mA/cm^2^, at the beginning of electrolysis, the heat generated by the system is more than the heat lost. Therefore, the temperature of the electrode liquid gradually increases. With the increase in temperature, heat dissipation is accelerated, and the system heat generation and heat dissipation gradually reach a balance, so the curve of electrode liquid temperature changing with electrolysis time presents a platform. The higher the current density is, the faster the heat is generated and the faster the temperature rises. When the current density is 67 mA/cm^2^, the current is too small and the heat generated is relatively small. In the whole process of electrolysis, the heat generated by the system is always equal to the heat lost. Therefore, the temperature of the electrode liquid will not rise but will maintain the ambient temperature.

Figure 9d shows the change curve of electrode liquid temperature in the process of electrolysis for 5 h, and the curve shows an overall trend of increase. When the electrolysis time was ~80 min, the temperature rose rapidly, because the system temperature was lower at this time, and the temperature difference between the system and the ambient temperature was small, and the heat loss was relatively slow. When the temperature of the system approached 40 °C, the increase in the temperature difference accelerated the loss of heat, thus reducing the rise rate of the system temperature. Within 80~270 min, the system temperature was in the stage of slow rise, and the electrode liquid temperature rose to 43.4 °C. After 270 min of electrolysis, the decrease in electrode liquid ion concentration led to an increase in solution resistance and an increase in cell voltage, which further accelerated the generation of heat. As a result, the temperature of the electrode liquid increased significantly. The highest temperature of the electrode liquid during electrolysis reached 51.9 °C. In the process of electrolysis, the temperature of the electrode liquid fluctuated several times. Especially in the last 90 min, the temperature of the electrode liquid generally increased, but it was very unstable and kept fluctuating. This is because the temperature field of the electrolytic system was not stable, and the heat distribution was uneven. In particular, there is a certain temperature difference between the electrolyte between the two electrodes and the electrolyte near the cell wall. Therefore, there was a transfer time in the process of heat transfer, especially when the temperature of the electrode liquid was higher; the slight disturbance of the solution might lead to an obvious fluctuation of temperature.

Although the high electrolyte temperature is conducive to the production of electrolytic products with good physical properties, the heat generated by the electrolytic system in the process of electrolysis will keep the electrolyte at 35~50 °C. Therefore, without additional heat, the electrolyte temperature achieved by the electrolysis at room temperature can meet the needs of the experiment.

### 3.3. Separation of Magnesium from Simulating Salt Lake Solution by Cationic Membrane Electrolysis

In the experiment, the diluted simulated brine was used as the anode liquid (A0) for electrolysis, the lithium chloride solution was used as the cathode liquid (C0), the current density was 300 mA/cm^2^, and the electrolysis time was 2, 4, 6, and 8 h, respectively. The compositions of the anode liquid and cathode liquid are shown in Table 2.

**Figure 10:**
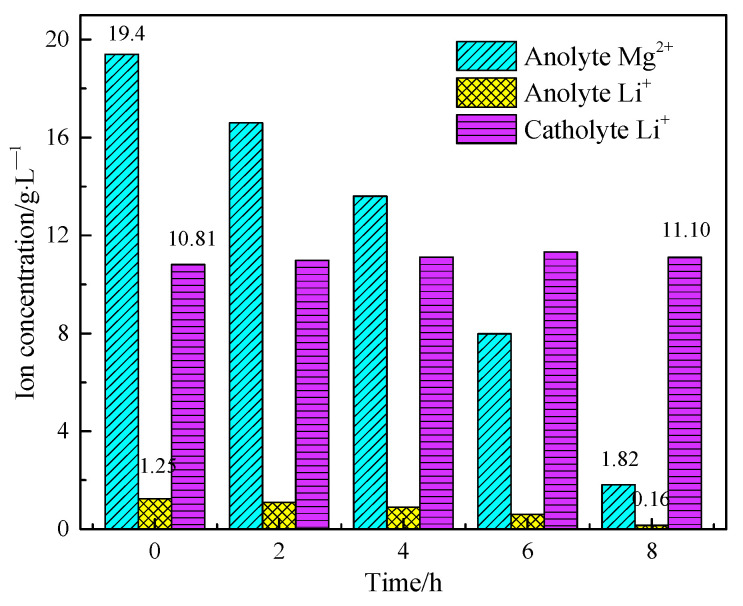
Ion concentrations in the anolyte and catholyte during electrolysis.

The volume of both cathode liquid and anode liquid was 300 mL. However, water molecules would also migrate through the ion exchange membrane during the electrolysis process, and there would be evaporation during the electrolysis process. Therefore, the cathode liquid volume would increase after electrolysis, while the anode liquid volume would decrease. The concentrations of Mg^2+^ and Li^+^ in the anode and cathode solutions before and after electrolysis were detected and compared, and the results are shown in Figure 10.

Figure 10 shows that with the extension of electrolysis time, the concentration of Mg^2+^ in the anode liquid decreased from 19.4 g/L to 1.82 g/L, and the extraction rate of magnesium reached 95.86%, successfully achieving the separation of magnesium and lithium in the salt lake and the utilization of magnesium. The concentration of Li^+^ in the anode liquid decreased from 1.25 g/L to 0.16 g/L, and the transfer proportion of Li^+^ from anode liquid to cathode liquid reached 88.31%. The composition and purity of magnesium hydroxide products produced by electrolysis of salt lake brine are shown in Table 3. The purity of magnesium hydroxide can reach about 99.5% and the residue of sodium and potassium in the magnesium hydroxide product is very low.

### 3.4. Extraction of Lithium from Cathode Liquid of Separating Magnesium

**Figure 11:**
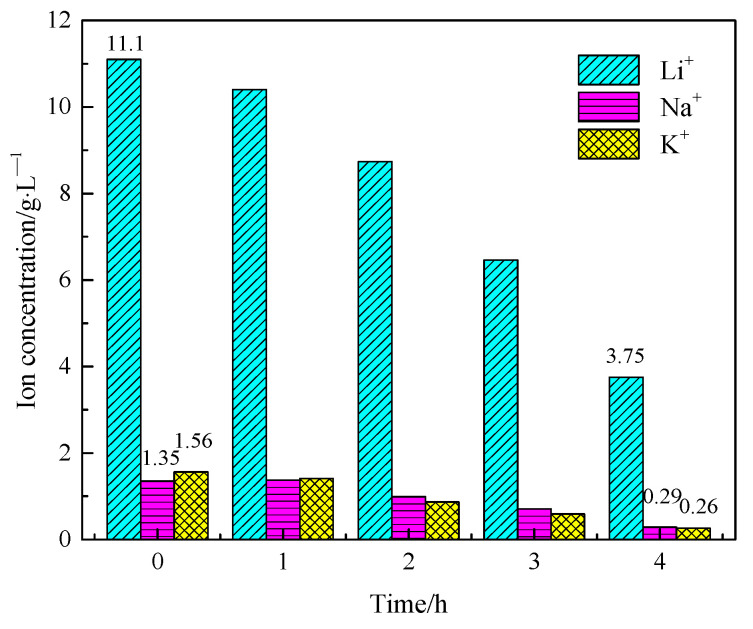
Concentration of cations in anode liquids at different electrolysis times.

The lithium-rich cathode solution extracted the magnesium was used as an electrolyte to prepare lithium carbonate. The experimental conditions were as follows: current density was 400 mA/cm^2^, CO_2_ flow was 0.5 L/min, and electrolysis time was 1, 2, 3, and 4 h, respectively. The changes in cationic concentration in the anode liquid with the electrolysis time are shown in Figure 11. The Li^+^ concentration of the anode liquid decreased from 11.1 g/L to 3.75 g/L, and the lithium extraction rate reached 67.46%. The element composition and purity of lithium carbonate are shown in Table 4. The purity of lithium carbonate can reach 99.98%, and the separation and extraction of lithium have been successfully realized.

**Figure 12:**
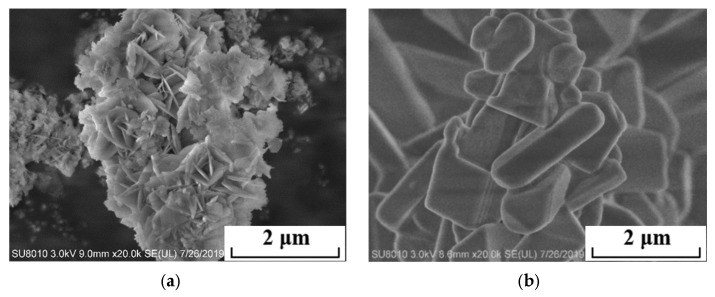
SEM images of the magnesium hydroxide (**a**) and lithium carbonate (**b**) products separated from the simulated brine.

The micromorphology of the electrolytic products magnesium hydroxide and lithium carbonate produced by two-step electrolysis is shown in Figure 12. Magnesium hydroxide is lamellar with a larger particle size and is easy to filter and separate. Lithium carbonate has a short-rod or granular shape.

## 4. Conclusions

The membrane electrolysis method proposed in this study enables effective treatment of high-magnesium salt lake brine, achieving efficient separation of magnesium and lithium while simultaneously producing magnesium hydroxide and lithium carbonate. This work systematically investigated the influence of electrolysis temperature on the physicochemical properties of Mg(OH)_2_ obtained via membrane electrolysis, and examined the variation in electrolyte temperature in relation to other factors, including electrolyte concentration (anolyte concentration 150 g/L, catholyte concentration 70 g/L), current density (201 mA/cm^2^), and processing time (2 h). Based on the results, direct electrolysis at room temperature was identified as a feasible operational condition. Experimental results demonstrated that during the preparation of magnesium hydroxide by membrane electrolysis, the extraction rate of magnesium reached 95.86%, with the resulting Mg(OH)_2_ attaining a purity of 99.5%. In the subsequent preparation of lithium carbonate from the magnesium-depleted cathode solution, a lithium recovery rate of 67.46% was achieved, and the final Li_2_CO_3_ product purity exceeded 99.98%. This study demonstrates a viable approach for the comprehensive utilization of high Mg/Li ratio salt lake brines, particularly in enabling the resource-oriented recovery of magnesium, showing significant research value and promising application potential.

## Figures and Tables

**Table 1:** Ion concentrations in the initial brine and evaporated liquids [3].

Ions/g·L^−1^	Li^+^	Mg^2+^	Na^+^	K^+^	Cl^−^	SO_4_^2−^	B_2_O_3_
Initial	0.3–5	8–22	68–114	6.7–14	144–231	3–47	0.8–17
Evaporated	2–8	100–120	0.3–3	0.2–1.8	300–340	23–54	10–43

**Table 2:** The compositions of the anode liquid and cathode liquid [3].

g/L	Li^+^	Na^+^	K^+^	Mg^2+^	Cl^−^	B	SO_4_^2−^
A0	1.25	1.39	1.53	19.4	57.1	1.00	10.6
C0	10.81	-	-	-	54.19	-	-

**Table 3:** The composition and purity of magnesium hydroxide.       Wt.%.

Time/h	Mg	Li	Na	K	Cl	B	SO_4_^2−^	Mg(OH)_2_
2	41.4	0.011	0.015	<0.001	0.12	0.13	0.009	99.715
4	41.7	0.02	0.016	0.001	0.16	0.12	0.007	99.676
6	40.3	0.035	0.088	0.003	0.13	0.24	0.018	99.496
8	39.7	0.11	0.025	0.012	0.21	0.12	0.007	99.516

**Table 4:** The composition and purity of lithium carbonate.        Wt.%.

Time/h	Li	C	Na	K	Cl	Li_2_CO_3_
1	18.4	16.3	0.004	0.004	0.008	99.984
2	18.2	16.0	0.004	0.004	0.004	99.988
3	18.3	16.1	0.004	0.005	0.003	99.988
4	18.6	15.8	0.008	0.004	0.007	99.981

## Data Availability

The original contributions presented in this study are included in the article. Further inquiries can be directed to the corresponding author.
